# Neonatal transport practices in Ibadan, Nigeria

**DOI:** 10.11604/pamj.2016.24.216.8651

**Published:** 2016-07-12

**Authors:** Muhydeen Abiodun Abdulraheem, Olukemi Oluwatoyin Tongo, Adebola Emmanuel Orimadegun, Olukayode Felix Akinbami

**Affiliations:** 1 Department of Paediatrics, University College Hospital, Ibadan, Nigeria; 2 Department of Paediatrics, College of Medicine, University of Ibadan, Ibadan, Oyo State, Nigeria; 3 Institute of Child health, College of Medicine, University of Ibadan, Ibadan, Oyo State, Nigeria; 4 Department of Paediatrics, College of Health Sciences, Niger Delta University, Bayelsa State, Nigeria

**Keywords:** Neonates, transport, Ibadan, Nigeria

## Abstract

**Introduction:**

Neonatal transport involves moving sick neonates in optimal conditions to ensure good outcomes. It is well organized in most developed countries but receives little attention in developing countries where the highest burden of neonatal mortality exists and a large number of newborns require referrals daily for better medical care. This study sought to evaluate the modes of transport, pre- and intra-transport care of neonates referred to the University College Hospital (UCH), Ibadan, Nigeria.

**Methods:**

The methods of transporting 401 neonates presenting to the children’s emergency room of the hospital were evaluated as well as the care the babies received during transport. Categorical variables were compared using the Chi square test while continuous variables were compared by the student t-test.

**Results:**

About a third presented in the first 12hours and 85% in the first week of life, all from within 80km radius. There were 67.1% term, 31.4% preterm and 1.5% post-term neonates, all without prior communication. The modes of transport included private vehicles (43.9%), commercial vehicles (40.6%), motorcycles (9.0%), ambulance (4.0%) and on foot (2.5%). Only 3 (0.7%) were transported in incubators and none in KMC position. Only 42.0% had referral letters and 7.0% were accompanied by medical personnel. Materials available during transport included Ambubags (3.7%), oxygen (3.5%) and some drugs (3.5%). Events during transport were apnoea, 4.7%, vomiting 1.0%, reduced activity 16.2% and seizures 13.7%. 19 (4.7%) neonates were dead on arrival. Pre-transport care included resuscitation (18.2%), intravenous fluid/feeding (24.4%) and supplemental oxygen (14.0%).

**Conclusion:**

Neonatal transport practices in Ibadan, Nigeria are abysmal with associated high mortality.

## Introduction

Transportation of sick neonates has a direct relationship with morbidity and mortality [1]. The concept of neonatal transport is a well recognised component of neonatal intensive care which is at various levels of development in technologically advanced countries. It demands professionalism and planning, and should only be undertaken in close cooperation with the unit receiving the infant [2]. The essence of neonatal transport medicine is to keep the infant stable and, preferably, improve the clinical status of the infant or at least, ensure it is not worse off on arrival at the receiving hospital [3]. The principle of neonatal transport includes; adequate preparation and stabilisation of the baby to be referred, communication with the receiving facility, and provision of standard care similar to that obtained in a neonatal intensive care unit during transfer. In the ideal setting, a baby should be stabilised in the intensive care unit (ICU) at the referring hospital, and transported by dedicated medical and paramedical staff in a mobile ICU to the referral hospital [3]. Previous studies have shown for example that, hypothermia, hyperthermia and hypoglycaemia resulting from poor transportation methods and care significantly increase neonatal mortality rate [4, 5].

In Nigeria, the entire process of neonatal transport which has the potential of impacting on morbidity at admission has received little or no attention. There are studies from Nigeria showing high prevalence of point of admission hypoglycaemia or hypothermia but no information on their transport to the hospital [6, 7].

This study, therefore, sought to describe the communication, pre- and intra-transport care, and transport facilities available to referred neonates in Ibadan and their association with immediate morbidity in order to determine areas of inadequacy and propose methods of improvement.

## Methods

It was a prospective and descriptive cross-sectional study conducted at the University College Hospital, (UCH) Ibadan. The UCH is a tertiary health facility that receives referrals from all other levels of health care in Oyo State of Nigeria and its environs. All referred neonates are first seen at the Children Emergency unit and given immediate care before admission into the neonatal wards. It involved enrolment of consecutive outborn `neonates presenting at the University College Hospital (UCH), Ibadan from August 2012 to February 2013. However, neonates with lethal congenital anomalies such as anencephaly were excluded from the study.

A structured case record form was used to obtain information relating to conditions pre-transport, intra-transport and at presentation. Some of the pre-transport information obtained included; sources of referral, birthweight, gestational age, method of resuscitation, duration of stay before referral, care given before referral and if there was any referral note. Intra-transport information taken included; types of vehicle, availability of emergency kit, method(s) of providing warmth and monitoring during transport. Other information included were presence of accompanying medical personnel, events during transport and the distance travelled and total duration of transport. All neonates brought in dead were also recruited. Information on resuscitation and the care given before referral was obtained from caregivers. The STABLE protocol, an acronym for sugar control, temperature control, airway maintenance, blood pressure, laboratory work, and emotional support for the family was used as basis for expected pre-transport care [8]. For this study, emotional support was taken as all forms of non medical support given to the parents at the sources of referral or during transportation to ameliorate the emotional distress the caregivers may be undergoing which included empathy, counselling regarding explanation of the diagnosis given to the caregivers or the patients from referring centres.

Also, the referring health facilities were visited to determine the pre-transport condition of the neonates, pre-transport care, transport facilities available to neonates, availability of equipment such as transport incubator, ventilator, SpO2 monitor, essential drugs for resuscitation, neonatal bag-mask. The distances between the referring health facilities and UCH were estimated using the odometer of the investigator’s car. On admission, detailed clinical assessment was carried out for each baby using the hospital standard protocol for the care of newborns.

Ethical approval: the study protocol was approved by the joint University of Ibadan/UCH’s Ethical Review Committee (Approval Number: UI/EC/12/0085). Participation in the study was voluntary and written informed consent was obtained from the mothers or caregivers of the neonates.

## Results

### Socio-demographic characteristics

A total of 411 eligible babies presented during the study period but 10 parents declined enrolment. Of the 401 newborns, 14(3.5%) were triplets, 37 (9.2%) twins and 350 (87.3%) singletons with 379 mothers. Eleven and one mothers respectively did not present with the complete set of twins and triplets. About 85% of the neonates presented in the first week of life. One-third (135; 33.7%) presented within the first 12 hours of life, 50 (12.5%) presented at 12 – 23 hours, 36 (9.0%) at 24 – 48 hours of life, 117 (29.2%) 2 – 7 days of life and 15.7% (n = 63) after 7 days of life. The mean age of the mothers was 29.5 ± 5.6 years. Almost half (165; 43.5%) belonged to the middle socio-economic class III, 29.6% belonged to the lower class IV, 4.5% class V while 4.7% and 17.7% belonged to the upper classes I and II, respectively.

### Gender, gestational age and weight of the neonates

There were 234 (58.4%) males, 166 (41.4%) females and 1 (0.2%) neonate with XY DSD. Two hundred and sixty-nine (67.1%) were term, 126 (31.4%) preterm and 6 (1.5%) post-term neonates. The birth weight of the neonates were neither known by the informants nor written in the referral letters in 60.8% of cases. Of the 157 whose birth weights were known, 20.7% were normal weight (2.5 – 3.9 kg), 15.7% were low birth weight (<2.5 kg) and 2.7% weighed >4.0 kg. However, at the emergency ward, weight of the neonates at presentation ranged from 0.70 – 4.60 kg with a mean of 2.52 ± 0.89 kg.

### Places and modes of delivery

Majority (228; 56.9%) of the neonates were delivered in General Hospitals and private hospitals. One-fifth of the neonates were either delivered at home or in mission/traditional birth attendants’ homes while 10.5% (n = 42) and 7.5% (n = 30) were delivered at Primary Health Centres (PHCs) and tertiary centres respectively. Two (0.5%) of the neonates were delivered in the cars conveying the mothers to the hospital of booking and one neonate (0.2%) was found abandoned by the road side. The majority (79.7%) of neonates were delivered per vagina. Other modes of delivery were elective CS (7.7%), emergency CS (12.1%), vacuum extraction (0.3%) and forceps (0.3%).

### Sources of referral and accompanying family/health workers

Seventy one (17.7%) were self-referrals from home while 330 (82.3%) babies were referred from various hospitals. The referred babies came from 87 different hospitals. Almost 70% of the neonates were referred from general hospitals/private hospitals. However, 72 (21.8%) of the referrals from hospitals/maternity centres were secondary referrals i.e. had been to one or more hospitals before arriving at the UCH. Four of the babies (1.0%) were transferred from the Accident and Emergency unit of the UCH having been delivered there before the mothers could be transferred to the labour ward.

The neonates were accompanied by the following caregivers: 99 (25.0%) by both mothers and fathers, 126 (31.4%) by mothers, 154 (38.4%) by fathers and 22 (5.5%) were brought by the relatives without either of the parents. Only 28 (7.0%) neonates were accompanied by a nurse or other health professional and of these, 4 (1.0%) were from the Accident and Emergency unit of the UCH, 5 (1.2%) from other tertiary hospitals, 17 (4.2%) from general/private hospitals, and only 2 (0.5%) were accompanied by a Community Health Extension Worker (CHEW) from PHC.

### Distance travelled

The distance travelled by the babies from point of last referral to UCH varied from 0.5km to 80km, with a mean of 10km but majority 245 (61.1%) came from within 10km radius. One hundred and forty eight (36.9%) travelled between 10km and 20km while only 8 (2.0%) travelled between 20km and 80km.

### The means of transportation

Only 16 (4%), neonates were brought in their hospital ambulances with only 3 (0.7%) transported in incubators. Others were brought in private vehicles (n = 176; 43.9%), taxis (n = 116; 28.9%), motorcycle (n = 36; 9%), tricycle (n = 7; 1.7%), commercial buses (n = 40; 10%) and adults who trekked from referral centres (n = 10; 2.5%). The modes of carrying neonates in the various vehicles were “arms of the caregivers” (n = 252; 62.8%) and strapping to the caregivers’ back (n = 142; 35.4%) and the 4 (1%) who were delivered at the Accident and Emergency unit of the UCH were transferred in cots. All (n = 401; 100%) the neonates were wrapped with various types of clothing. Only 1 (0.2%) neonate from a private hospital had a well wrapped hot water bottle applied. Nobody employed kangaroo position to transport the babies.

While 60% of the babies came from centres with ambulance services, only 4% were brought in hospital-owned ambulances. In 2.3% of the health facilities, the ambulances were non-functional while in others they did not just consider it necessary. Only one of such ambulances was suitable to carry transport incubators. The various facilities available at the referring hospitals are as listed in Table 1.

**Table 1:** Facilities/equipment available at sources of referral

Equipment	Source of referral								Total
	Maternity		PHC		GH/Private		Tertiary		
	n	%	n	%	n	%	n	%	N
Ambubag	0	0	13	5.1	219	86.6	17	6.7	253
Supplemental oxygen	0	0	14	5.2	243	87.7	20	7.2	277
Glucometer	3	1	16	5.2	270	88.5	16	5.2	305
Pulse Oximeter	0	0	0	0	41	71.9	16	28.1	57
Incubator	0	0	0	0	184	92.0	16	8.0	200
Transport incubator	0	0	0	0	8	33.3	16	66.7	24
Ambulance	0	0	8	3.3	217	90.0	16	6.6	241
Laboratory services	0	0	15	6.3	205	86.9	16	6.8	236

Emergency preparedness and clinical events during transport About 93% had no medical equipment/resuscitation kits available during transport. Of the 28 (7%) neonates who were accompanied by medical personnel and transported with medical equipment, materials available during transport were Ambubags (n = 15; 3.7%), oxygen cylinders (n = 14; 3.5%) (some in private vehicles), drugs such as antibiotics, hydrocortisone, adrenaline (n = 14; 3.5%), and 11 (2.7%) had other materials such as monitoring devices – pulse oximeters, glucometers, and medical consumables. The events that occurred during transport as reported by the accompanying caregivers and/or medical personnel were apnoea (n = 19; 4.7%), vomiting (n = 4; 1.0%), reduced activity (n = 65; 16.2%) and seizures (n = 55; 13.7%).

### Referral letters and communication with UCH prior to referral

Sixty five percent of the babies had referral letters while 17.3% were verbally referred from these hospitals without any letters given to them. Only 51% of the referral letters had complete information. The caregivers could not produce the letters immediately in 5.3% of cases. One baby came with the prototype referral letter for PHCs. There was no prior communication with UCH from any of the referring centres and no formal communication from UCH with any of the referring centres.

### Pre-transport and Intra-transport care

The information on pre-transport care provided at the referring hospitals indicated that 73 (18.2%) of the neonates received some form of resuscitation and 56 (14.0%) were given supplemental oxygen. Intravenous fluids or feeding with cup and spoon and/or breast feeding in 98 (24.4%), blood sugar was checked at least once in 26 (6.5%), and temperature was checked and documented in 54 (13.5%) of the neonates. Evidence of emotional support for families of the neonates was obtained in 45 (11.2%) and 63 (15.7%) of the neonates were given essential drugs such as adrenaline, dopamine and hydrocortisone pre-transport. The pre-transport care and intratransport given at the different healthcare facilities are as shown in Table 2 and Table 3.

**Table 2:** Pre-transport care/stabilisation provided at the different sources of referral

Care given^+^	Centre of referral												Total
	Home		TBA		Maternity		PHC		GH/Private		Tertiary		
	N	%	N	%	n	%	N	%	n	%	n	%	N
Resuscitation	1	1.4	0	0	3	4.1	2	2.7	64	87.7	3	4.1	73
IVF/feeding	20	20.4	0	0	2	2.0	5	5.1	63	64.3	8	8.2	98
Supplemental oxygen	0	0	0	0	1	1.8	3	5.4	50	89.3	2	3.6	56
Blood sugar checked	0	0	0	0	1	3.8	2	7.7	20	76.9	3	11.5	26
Temperature checked	4	7.4	0	0	2	3.7	2	3.7	42	77.8	4	7.4	54
Emotional support of parents	0	0	0	0	2	4.4	2	4.4	37	82.2	4	8.9	45
Drugs	7	11.0	0	0	2	3.2	4	6.4	46	73.0	4	6.4	63

**Table 3:** Intra-transport care provided by different sources of referral

Care given^+^	Centre of referral												Total
	Home		TBA		Maternity		PHC		GH/private		Tertiary		
	N	%	N	%	n	%	n	%	N	%	n	%	N
IVF	0	0	0	0	0	0	0	0	8	80	2	20	10
Breastfeeding	25	37.9	1	1.5	1	1.5	3	4.5	31	47.0	5	7.6	66
Supplemental oxygen	0	0	0	0	0	0	0	0	12	85.7	2	14.3	14
Oxygen saturation checked	0	0	0	0	0	0	0	0	3	100	0	0	3
Sugar checked	0	0	0	0	0	0	0	0	1	100	0	0	1
Temperature checked	1	14.3	0	0	0	0	0	0	6	85.7	0	0	7
Drugs	0	0	0	0	0	0	0	0	10	71.4	10	28.6	14

While 135 (33.7%) had their breathing observed, only 3 (0.7%) had their oxygen saturation measured. None of the caregivers of the neonates had evidence of emotional support during transport.

### Outcome of the transported neonates and provisional diagnosis at presentation

Nineteen babies (4.7%) were dead on arrival and all of them had been through multiple referrals. Among the 382 neonates who were brought in alive, provisional diagnosis at presentation included: severe perinatal asphyxia (n = 192; 47.9%), neonatal sepsis (n = 152; 38.0%), prematurity (n = 126; 31.4%), neonatal jaundice (n = 105; 26.2%), meconium aspiration syndrome (n = 8; 2.0%), surgical cases (n = 29; 7.2%) and others (n = 18; 4.5%). The surgical conditions included omphalocoele, spinal bifida, cystic hygroma, tracheoesophageal fistula and anorectal malformations. The other diagnoses included neonatal tetanus, cholestatic jaundice and various congenital anomalies – posterior urethral valve, Prune belly syndrome and Turner’s syndrome.

There was a significant association between intratransport mortality and gestational age categories, 8.7% (11/126) of preterm compared with 2.9% (8/275) of term neonates died in the course of transportation (p = 0.01). The odds of death before arrival in the emergency unit was 3 times higher among preterm than term neonates (OR = 3.19, 95% CI 1.25, 8.14). There was no significant association between gender and mortality; 5.1% (12/234) male compared with 4.2% (7/166) female neonates died during transport (p = 0.673). Table 4 shows the association between mortality before arrival UCH and the pre-transport or intra-transport care received. Mortality rate was not significantly different between neonates transported over a distance of more than 10 km (5.1%, 8/156) and those moved ?10 km (4.5%, 11/245); p = 0.776.

**Table 4:** Association between mortality on arrival in the and the Pre-Transport and intra-transport care

	Pre-transport			Intra-transport		
	BID	Survived	p	BID	Survived	p
**Resuscitation**						
Yes	3 (4.1)	70(95.9)	1.000	-	-	
No	16 (4.9)	312(95.1)		-	-	
**IVF/Feeding**						
Yes	2 (2.0)	96 (98.0)	0.178	0 (0.0)	76 (100.0)	1.000
No	17 (5.6)	286 (94.4)		19 (4.8)	306 (94.2)	
**Oxygen**						
Yes	2 (3.4)	54 (96.6)	1.000	0 (0.0)	14 (100.0)	1.000
No	17 (5.0)	328 (95.0)		19 (4.8)	368 (95.1)	
**Sugar monitored**						
Yes	1 (3.7)	25 (96.3)	1.000	0(0)	1(100)	1.000
No	18 (4.8)	357 (95.2)		19 (4.8)	381 (95.2)	
**Temperature check**						
Yes	1 (1.8)	53 (98.2)	0.492	0 (0.0)	7 (100.0)	1.000
No	18 (5.2)	329 (94.8)		19 (4.8)	375 (95.2)	
**Emotional support**						
Yes	1 (2.2)	46 (97.8)	0.710	-	-	
No	18 (5.1)	336 (94.9)		-	-	
**Drugs**						
Yes	1(1.6)	62 (98.4)	0.333	-	-	
No	18 (5.3)	320 (94.7)		-	-	

## Discussion

The importance of transporting neonates under controlled conditions for better outcomes cannot be overemphasized. Though in-utero transfer is the safest form of neonatal transport [9–14], however, preterm delivery, perinatal illnesses and congenital malformations may not always be anticipated with continuous need to transport babies after delivery even in resource limited settings [14]. These babies are often critically ill, and their outcome will be partly dependent on the effectiveness of the transport system [15].

Nigeria has a high annual neonatal mortality rate (NMR) of 37 per 1000 live births and represents the highest number of newborn deaths in Africa and the second highest in the world [16, 17]. Reducing the burden of neonatal deaths is a pressing issue. To achieve this reduction, a number of packages for improving newborn care such as integrated maternal, newborn & child health (IMNCH) strategy, community newborn care and essential newborn care (ENC) have been adopted and they all emphasize the need for prompt identification and referral of sick neonates upon recognition. An effective referral ought to be done in a manner that is safe and will enhance survival of the sick neonate.

As shown in this study, the spectrum of conditions in the referred babies included severe perinatal asphyxia, prematurity and surgical cases which definitely required specialized care available only at secondary and tertiary facilities in Nigeria. Though no formal regionalization of perinatal care exists in Nigeria but the secondary and tertiary facilities where specialized perinatal/neonatal are quite few and babies have to move from peripheral centres to access such care. The distance travelled by babies in this study was 0.5km – 80km which ordinarily is not too far if appropriate transport services are available. In the light of this, irrespective of the level of human and material resources available in our setting, the principle of neonatal transport must be adhered to, utilising available and appropriate technology. The transport of neonates in this setting is definitely suboptimal in terms of poor pretransport stabilization, communication and the actual transport. The post-resuscitation and pre-transport care of sick babies as described in the “STABLE programme” emphasises: sugar control, temperature control, airway maintenance, blood pressure, laboratory work, and emotional support for the family. The STABLE provides a comprehensive approach to optimising care prior to transport.[8] These are usually neglected or not appropriately done by healthcare workers in many developing countries. That was also typified in this study as less than 20% of the babies had some form of resuscitation and barely a quarter had attention paid to glucose control by way of intravenous fluids or feeding before referral. There was no prior communication with UCH in any of the babies to give opportunity for adequate preparation for the arrival of these babies. Also no information was available on the clinical stability of the babies from the referring centres. Referral letters were unavailable in many instances and where available, the contents were largely inadequate similar to what was reported in a previous study in the same centre [18]. These show that no considerations were given to the suitability of the babies for the transport and coupled with all the other inadequacies with the transport, it is not surprising that 4.7% were brought in dead and it is not unlikely that many more had died during transport and were never brought to UCH. The practice therefore negates the whole essence of referring the babies.

Inadequacy of initial resuscitation, failure to provide warmth and poor glycaemic control, unsafe and unorganised transport practices as well as failure to write detailed referral notes to the receiving doctors in neonatal care units have been recognised as barriers to effective management of the sick neonates in developing countries [4]. With inadequate pre- and intra-transport stabilisation the condition of most referred neonates on arrival at the neonatal care facility, therefore, can be much worse than their pre-transport status.

Majority of the babies in this study were transported by various means including open tricycles which are definitely not suitable for such purposes and without accompanying health care workers to monitor them during transport. Studies in another developing country also revealed such dismal facilities for neonatal transport [19, 20]. Many neonates are daily transported with any available vehicle which often takes long hours and without any medical personnel accompanying such transport. Many of the babies transported arrived cold, blue and hypoglycemic, and as much as 75% of the babies transferred this way have serious clinical implications [21–23]. Studies have shown that even in settings with neonatal transport services, in the presence of less experienced personnel accompanying neonatal transport, the risk of adverse events on such transports can be greater than with well-equipped and trained personnel [24–26]. As shown in this study, there were hardly any health care workers accompanying the babies and in the 7% that were accompanied, only about half were equipped with basic facilities such as bag and mask device or oxygen in the event of any emergencies during transport.

The drive to reduce neonatal mortality in developing countries by training primary health care workers in various aspects of essential newborn care and particularly recognition of danger signs and referral has to be complimented by teaching safe transport as well. Even in the absence of sophisticated neonatal transport facilities, evidence based low cost interventions such as kangaroo mother care position can be more effectively utilised for the transport of such babies with those who can tolerate oral feeds fed with expressed breastmilk during transport. This has been proposed in the national guideline by the Federal Ministry of Health [27] for use in newborn transport apart from basic care in the hospital and community though it was not utilized in any of the referrals in this study.

It is crucial to start advocating for the development of neonatal transport services at least starting from ground travel and any other form of affordable and sustainable safe transport in Nigeria and other developing countries if referred neonates are to arrive at tertiary facilities in optimal condition. It is also essential to begin to incorporate neonatal transport into the training curriculum of medical schools and training institutions for health workers in developing countries. Formal training in transportation skills is provided in most US paediatric training programmes, but, currently, it forms only a small part of the undergraduate or postgraduate training in most African schools [2]. This study was partly conducted to beam searchlight on neonatal transport in Nigeria so that it can begin to receive necessary attention.

## Conclusion

The practice of neonatal transport in Ibadan, Nigeria is far from ideal and is associated with significant mortality of the referred neonates. No aspect of referral was adequate from communication to preparation/stabilisation for transport and the actual transport itself. It is recommended that in utero transfer be utilised more especially in cases of preterm deliveries which constituted about a third of the subjects pending such a time when neonatal transport will become recognised and organized in this setting.

### What is known about this topic

- Neonatal transport is well organized in developed countrie; there is usually resuscitation, stabilization and communication prior to transportation of newborns to other centres for further care;
- The situation during transportation of sick neonates is usually controlled such that there is ongoing monitoring and care equivalent at least to the care available at the referring centre;
- Information on neonatal transport in subSaharan Africa and even Nigeria is not available in literature.

### What this study adds

- Transport of sick neonates in Nigeria is not organized;
- Inadequate pretransportation communication and stabilization is prevalent;
- Neonatal transport practices in Ibadan, Nigeria are suboptimal and are associated with poor outcomes (poor vital signs and mortality on admission).
